# Role of Polyamines in Asthma Pathophysiology

**DOI:** 10.3390/medsci6010004

**Published:** 2018-01-06

**Authors:** Vaibhav Jain

**Affiliations:** 1 Centre of Excellence for Translational Research in Asthma & Lung Disease, CSIR-Institute of Genomics and Integrative Biology (CSIR-IGIB), Mall Road, Delhi 110007, India; jain.vaibhav@igib.in or vaibhavj85@outlook.com; Tel.: +91-112-766-2580; Fax: +91-11-2766-7471; 2 Academy of Scientific and Innovative Research (AcSIR), Chennai 600113, India

**Keywords:** spermine, spermidine, putrescine, polyamine metabolism, mast cells, eosinophils, neutrophils, M2 macrophages, airway smooth muscle cells

## Abstract

Asthma is a complex disease of airways, where the interactions of immune and structural cells result in disease outcomes with airway remodeling and airway hyper-responsiveness. Polyamines, which are small-sized, natural super-cations, interact with negatively charged intracellular macromolecules, and altered levels of polyamines and their interactions have been associated with different pathological conditions including asthma. Elevated levels of polyamines have been reported in the circulation of asthmatic patients as well as in the lungs of a murine model of asthma. In various studies, polyamines were found to potentiate the pathogenic potential of inflammatory cells, such as mast cells and granulocytes (eosinophils and neutrophils), by either inducing the release of their pro-inflammatory mediators or prolonging their life span. Additionally, polyamines were crucial in the differentiation and alternative activation of macrophages, which play an important role in asthma pathology. Importantly, polyamines cause airway smooth muscle contraction and thus airway hyper-responsiveness, which is the key feature in asthma pathophysiology. High levels of polyamines in asthma and their active cellular and macromolecular interactions indicate the importance of the polyamine pathway in asthma pathogenesis; therefore, modulation of polyamine levels could be a suitable approach in acute and severe asthma management. This review summarizes the possible roles of polyamines in different pathophysiological features of asthma.

## 1. Introduction

Polyamines were discovered late in the 16th century as crystals in human semen by Antonie van Leeuwenhoek [1]. Now, polyamines are defined as positively charged aliphatic alkylamines which are natural supercations carrying two, three and four positive charges on putrescine (H_3_N^+^(CH_2_)_4_^+^NH_3_), spermidine (H_3_N^+^(CH_2_)_4_^+^NH_2_(CH_2_)_3_^+^NH_3_) and spermine (H_3_N^+^(CH_2_)_3_^+^NH_2_(CH_2_)_4_^+^NH_2_(CH_2_)_3_^+^NH_3_), respectively, at physiological pH [2]. These positively charged polyamines interact with negatively charged molecules in a cell such as DNA, RNA, proteins, and proteoglycans; hence, polyamines are involved in a myriad of biological processes [3,4]. Broadly, they are involved in the processes that play important roles in cell survival or death pathways, such as proliferation, differentiation, autophagy, apoptosis and translation [5,6,7,8]. Interestingly, depending on the concentration of polyamines and cell type, a cell may undergo proliferation, differentiation, apoptosis or quiescence. Generally, polyamines are thought to be required for cell proliferation and growth and high levels of polyamines have been observed in multiple cancerous conditions [9]. In contrast, their levels decline with aging, where replicative senescence of cells plays an important role [6,10]. However, the effects of polyamines are contextual and depending on their concentrations and cell type, their effects may vary. Cells with high proliferation rates may need higher levels of polyamines than their optimal levels. However, in cells that are not directed for proliferation, supra-optimal levels of polyamines may lead to apoptosis, while sub-optimal levels of polyamines may lead to a quiescent state [8].

Asthma is a complex disease that is defined by chronic airway inflammation, remodeling and hyperresponsiveness. The pathogenesis of asthma is multifactorial and includes complex interactions between genetic and environmental factors leading to multiple phenotypes that differ in their clinical and physiological features as well as their response to therapy [11]. For example, two phenotypes of asthma include patients differing in their responsiveness to corticosteroid therapy. Unlike in corticosteroid-responsive asthmatics, where the airway inflammation is majorly eosinophilic, patients with severe asthma predominantly show neutrophilic airway inflammation and are poor responders to corticosteroid treatment [12]. This indicates that there is a need to discover alternative treatments for asthma, since existing treatments are not very effective for all severities of asthma. High levels of polyamines in the circulation of asthmatics with active symptoms and in the lungs of a murine model of asthma [13,14] indicates that polyamines could play an important role in respiratory diseases. Additionally, the interaction of polyamines with the immune and structural cells of asthma pathophysiology indicates that the polyamine pathway plays an active role in the asthmatic response.

This review summarizes the studies highlighting the importance of polyamines in asthma pathophysiology. The outcomes from various studies indicate that the polyamine pathway may play a decisive role and could be an important therapeutic target in different phenotypes of asthma such as acute and severe asthma.

## 2. Homeostasis of Polyamines

**Figure 1:**
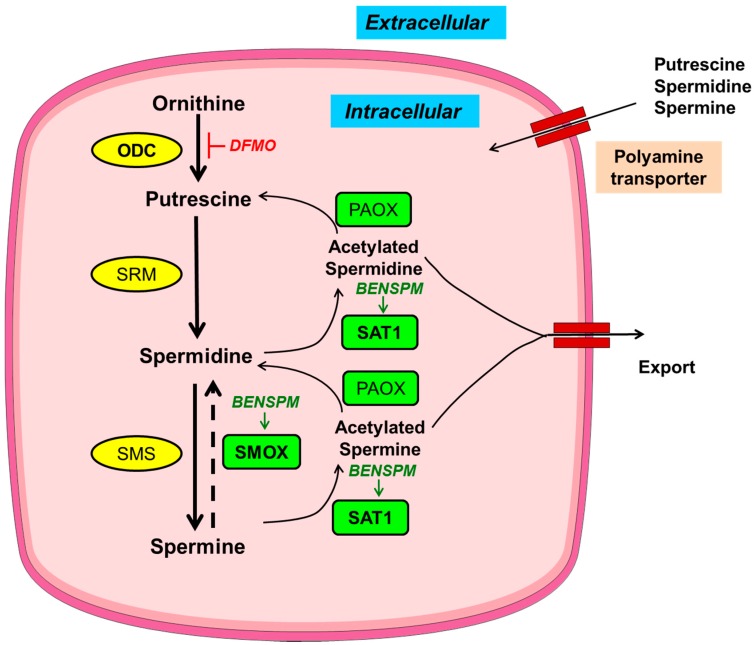
Schematic depiction of polyamine metabolism and transport. Anabolic and catabolic enzymes in yellow circles or green rectangles, respectively, with key metabolic enzymes in bold. Pharmacological inducer and inhibitor are italicized in green and red, respectively. Ornithine decarboxylase (ODC) synthesizes putrescine from ornithine; putrescine further gets converted into spermidine by spermidine synthase (SRM) and further to the largest polyamine spermine by spermine synthase (SMS). During catabolism, spermine can be back converted into smaller polyamines, i.e., spermidine and putrescine, by the concerted actions of spermidine/spermine N1-acetyltransferase (SAT1) and polyamine oxidase (PAOX) with an intermediate acetylation step mediated by SAT1; acetylated polyamines are either exported out of the cells or catabolized by PAOX. Spermine oxidase (SMOX), another enzyme of polyamine catabolism, directly converts spermine into spermidine without an intermediate acetylation step. In addition to de novo synthesis, polyamines can also be transported. BENSPM: bis(ethyl)norspermine; DMFO: 2-difluoromethylornithine.

The levels of polyamines are tightly regulated by their finely tuned metabolism and transport (Figure 1) [15,16]. Polyamine metabolism is a very specific and well-studied process in comparison to the poorly explored polyamine transport mechanism. Of note, there are pharmacological modulators available for the key enzymes of polyamine metabolic pathway. The modulators of polyamine metabolism are used either to alter the levels of polyamines or to study the importance of polyamine metabolic enzymes in a cell. Of the modulators of polyamine metabolism, the most widely used compounds are: 2-difluoromethylornithine (DFMO) and bis(ethyl)norspermine (BENSPM) (Figure 1). DFMO is an irreversible inhibitor of the polyamine anabolic enzyme, ornithine decarboxylase (ODC). DFMO reduces the levels of putrescine and spermidine [17]; however, DFMO is generally ineffective in bringing down the levels of spermine due to other compensatory mechanisms of polyamine uptake and incomplete inhibition of ODC by DFMO [18,19]. BENSPM is a spermine analogue that suppresses the polyamine biosynthetic enzyme ODC, while it potently induces the polyamine catabolic enzymes spermidine/spermine N1-acetyltransferase (SAT1) and spermine oxidase (SMOX) and depletes the natural polyamines pools, i.e., putrescine, spermidine and spermine. BENSPM also competes for the import of polyamines [20,21].

## 3. Asthma Pathophysiology

Asthma is a heterogeneous airway disease that is associated with chronic airway inflammation. Asthmatic patients show a history of wheezing, shortness of breath, chest tightness and cough, all with variations in intensity over time. Airway inflammation in asthma involves a complex interplay of structural and immune cells [22]. The immune component includes mainly leukocytes, both granulocytes and lymphocytes. Dendritic cells process and present antigens to T lymphocytes, resulting in the development of type-2 T helper cell (Th2) responses [23]. This Th2 immune response plays a dominant role in asthma pathophysiology. Th2 cells produce cytokines like interleukin (IL)-4, IL-5 and IL-13. These cytokines and Th2 cells together enhance allergic response by inducing several pathways [24]. IL-4 promotes immunoglobulin class switching from immunoglobulin G (IgG) to IgE isotype in B cells [25]. Additionally, IL-4 induces the expression of IgE receptors on mast cells and basophils, which bind IgE and subsequently release preformed mediators that are important in asthma pathology [26]. IL-13 is another key cytokine of asthma; it shows pleiotropic effects on multiple cells. IL-13 is involved in the class switching of immunoglobulin to IgE isotype and promotes the Th2 response, along with airway hyper-responsiveness and airway remodeling [27]. Interestingly, administration or overexpression of either the IL-4 or IL-13 cytokine alone, in naïve mice lungs, can generate key features of allergic airway disease [28,29]. IL-5 is an important cytokine for the survival, growth and activation of eosinophils, which promote innate and adaptive immune response during allergic airway inflammation [30]. In addition, macrophages play an active role in asthma. In asthma, monocytes differentiate into the M2 or alternatively activated (M2) macrophage (AAM) phenotype in a Th2-dominant microenvironment [31]. It has been shown that the abundance of these AAMs negatively correlates with the lung function of asthma patients [32]. 

In asthma, in addition to the immune cells, the active involvement of structural cells, i.e., of airway smooth muscle cells (ASMCs), fibroblasts and airway epithelial cells, have been explored. Remodeling of lung architecture in response to a Th2 environment increases the thickness of the sub-epithelial basement membrane in lung bronchus due to the proliferation of ASMCs and fibroblasts. Further, ASMCs and fibroblasts secrete collagen and other extracellular matrix molecules contributing to the thickness of the sub-epithelial region. Airway remodeling has been thought to contribute to airway hyper-responsiveness by increasing the stiffness of airways [33]. In addition to ASMCs and fibroblasts, current literature suggests that airway epithelium could play an active and causative role in asthma pathogenesis [34]. Airway epithelium is the first line of defense between lung and external environment. In asthma, airway epithelium has been reported to be damaged and is sensitive to apoptosis [35]. Since airway epithelium is exposed to the external environment, it encounters various environmental insults, allergens and antigens, which bring airway epithelial cells under stress. Injured or stressed airway epithelial cells secrete many factors such as cytokines. These cytokines, such as thymic stromal lymphopoietin (TSLP), IL-25 and IL-33, together called alarmins, are released by stressed airway epithelial cells. These alarmins can govern Th2 immune responses and other key features of asthma, such as airway hyper-responsiveness and airway remodeling [36]. Moreover, airway epithelial cells undergo mucus metaplasia and epithelial-to-mesenchymal transition, in which airway epithelial cells change their phenotype to mucus-producing goblet cells and a fibroblast phenotype, respectively, that contributes to airway wall remodeling [37]. Taken together, in asthma, immune and structural cells interact with each other, resulting in the disease pathophysiology; however, the cause and effect relationship between immune and structural component of asthma pathogenesis remains elusive.

## 4. Alteration of Polyamines in Asthma

The first report of polyamines in asthma was in the blood of asthmatic patients; the levels of polyamines were reported to be high in the blood of asthmatic patients, particularly during asthmatic attacks [13]. Additionally, elevated polyamine levels were reported in the lungs of a murine model of asthma [38]. In addition to the high levels in blood, the polyamine spermine was found to be significantly high in the bronchoalveolar lavage fluids of asthmatic patients, which is a direct reflection of lung microenvironment [14].

## 5. Polyamines Affect Immune Cells in Asthma

**Figure 2:**
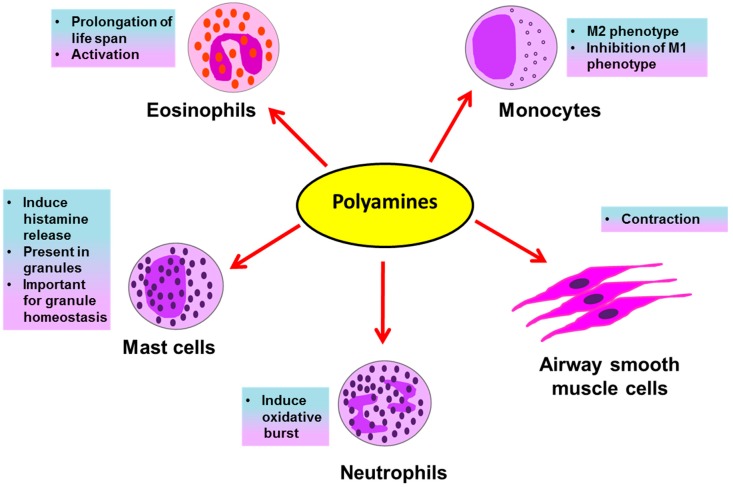
Possible effects of polyamines on immune and structural cells in asthma.

Eosinophils, neutrophils, basophils, mast cells and macrophages are all inflammatory cells involved in asthma pathology. The inflammatory response in allergic asthma is eosinophilic. However, along with eosinophilia, severe asthmatic patients show the involvement of a neutrophilic response [39]. Eosinophils and neutrophils are inflammatory granulocytes; they release several preformed inflammatory mediators stored in their secretory granules upon their activation in an allergic environment of the lungs. These mediators, such as histamines, leukotrienes, reactive oxygen species (ROS), and toxic granule proteins, induce asthmatic exacerbations and inflammation in the lungs by causing bronchoconstriction, tissue damage, airway inflammation and remodeling. Histamines are biogenic amines derived from the amino acid histidine and are present in mast cell granules; they induce bronchoconstriction, vasodilation and mucus secretion [40]. The generation of ROS by granulocytes in asthma is accompanied with tissue damage during airway inflammation [41]. Several oxidative products, like H_2_O_2_ and superoxide anions, are released that themselves cause damage and may further react with other molecules to generate damaging oxidative products such as leukotrienes, reactive nitrogen species, etc. Granulocytes also release leukotrienes, which are oxidative products of lipids and powerful bronchoconstrictors [42,43]. These mediators released by granulocytes are potential therapeutic targets for asthma. Polyamines affect immune cells during asthma in several ways (Figure 2), as briefly discussed below. 

### 5.1. Polyamines and Mast Cells

The association of high levels of polyamines with the functional response of immune cells was uncovered by Kurosawa et al., who demonstrated the effect of polyamines on mast cells. Polyamines, specifically spermine and spermidine at higher concentrations (1 mM) were demonstrated to induce the rapid release of histamine, a bronchoconstrictor, from mast cells (Figure 2). Additionally, at lower concentrations (0.1 mM) polyamines alone were ineffective; however, they did potentiate IgE-induced histamine release [44]. The mechanism behind this induction was speculated to be the ability of spermine and spermidine to activate the phosphatidylinositol kinase enzyme in the secretory granules, which play a crucial role in the release of histamine from mast cells [45]. Another report for mast cells came from a well-known interaction of polyamines and proteoglycans. The proteoglycan serglycin is very important for maintaining secretory granule structure and the packaging of secretory compounds like histamine and serotonin in mast cell granules. Interestingly, polyamines, specifically spermine and spermidine, were found to be present in the mast cell secretory granules and the concerted interactions of both, polyamines and serglycin, was found to be crucial for maintaining secretory compounds in granules. By using DFMO, polyamines were reduced in mast cell granules and it was found that the ultrastructure of mast cell granules was disrupted along with a disturbance in the homeostasis of secretory compounds; the morphology observed with DFMO treatment was similar to the morphology seen after depletion of serglycin, showing the importance of both polyamines and serglycin in granule morphology and function [46]. Recently a transporter, vesicular polyamine transporter (VPAT) was identified in the mast cell. VPAT was found to be responsible for the active storage and release of polyamines from the secretory granules of mast cells [47]. 

### 5.2. Polyamines and Eosinophils

In context of another important granulocyte in asthma pathology, the eosinophil, an interesting study demonstrated that polyamines, especially spermine (median effective concentration of 15 μmol/L), prolong normal life span of eosinophils [48] (Figure 2). In this study, spermine inhibited the normal apoptosis of eosinophils from normal subjects in addition to asthmatic subjects, thereby prolonging their life span in ex vivo culture. Spermine was the most active polyamine followed by spermidine; however, putrescine was ineffective in inducing survival of eosinophils. In this report, spermine was found to inhibit apoptosis linked to mitochondrial permeability transition (mPT), along with the activities of effector apoptotic caspases 3/7 and the initiator caspases 8 and 9. Further, spermine induced the expression of the adhesion molecule integrin, CD11b, on eosinophils. Though increased expression of CD11b on eosinophils is related with the capacity of their super oxide generation [49], spermine alone was not sufficient; however, it potentiated the oxidative burst induced by a bacterial chemotactic peptide, N-formyl-methionyl-leucine-phenylalanine (FMLP) peptide.

### 5.3. Polyamines and Neutrophils

In severe asthma, neutrophils play a crucial role. To study the role of neutrophils in asthma, neutrophils from the blood of asthmatics are stimulated with FMLP or phorbol myristate acetate (PMA) to induce an oxidative burst [50], followed by the addition of different chemicals to be tested for their effects on neutrophils. In one study, FMLP- and PMA-stimulated neutrophils from mild and severe asthmatics were tested for their steroid responsiveness. In contrast to mild asthmatics, neutrophils from severe asthmatics were unresponsive to treatment with the steroid prednisolone, in terms of reducing oxidative burst [51]. Interestingly, in a separate study on human neutrophils, the polyamines putrescine, spermidine and spermine (10–100 μM) induced respiratory burst (Figure 2) generated by FMLP, possibly by enhancing of the availability of exogenous Ca^2+^ [52].

### 5.4. Polyamines and Monocytes/Macrophages

Macrophages are important antigen-presenting cells in asthma and constitute the most abundant immune cells in the lung (approximately 70% of the total immune cells) [32]. In the asthmatic patient’s lung environment, monocytes majorly differentiate into AAMs [32,53]. These M2 macrophages are very important for the generation of the Th2 immune response in asthma. M2 macrophages are characterized by the increased expression of IL-4 and IL-13, which are very crucial cytokines in asthma pathogenesis. Interestingly, theses cytokines also promote the proliferation and differentiation of M2 macrophages in an autocrine manner [31]. Further, depletion of M2 macrophages in a murine model of allergic asthma was shown to attenuate inflammation and remodeling of airways [54]. A pivotal evidence of the interaction of polyamines and the Th2 response in asthma is evident from a study done in macrophages, where IL-4, a classical cytokine of allergic asthma, induced polyamine synthesis in macrophages and this production of polyamines orchestrated them into AAMs, while inhibiting the production of classically activated pro-inflammatory M1 macrophages [55] (Figure 2). Interestingly, the depletion of all polyamines after inducing polyamine catabolism using the drug BENSPM markedly reduced the expression levels of several genes or markers of AAMs, confirming a crucial role of polyamines in the polarization of AAMs. However, as the use of DFMO was not effective in reducing the polyamine spermine, it was used to uncover a set of genes modulated by the levels of putrescine and spermidine. The polyamines were needed as cofactors to induce the expression of several AAM genes induced by IL-4. Out of a total of 15 markers of AAM tested in this study, 9 markers were affected by the depletion of polyamines; the remaining unaltered markers were termed as polyamine-independent markers of AAMs. By using DFMO and BENSPM, this study exclusively determined the set of genes specifically induced by the individual polyamines putrescine, spermidine and spermine.

## 6. Polyamines and Structural Airway Smooth Muscle Cells

Along with inflammation, airway remodeling is a key feature of asthma pathology. Airway smooth muscle cells (ASMCs) are the major contributors in airway remodeling. A reversible contractility of the ASMCs leads to airflow limitation in asthmatic patients [56]. ASMC proliferation rate was found to be higher in asthmatics in comparison to ASMCs from normal individuals which leads to increase the numbers of ASMCs in airways, thereby thickening the airway wall [57]. ASMCs express smooth muscle-specific contractile proteins like α-smooth muscle actin, smooth muscle-specific heavy chain and calponin, myosin light chain kinase and others [58]. These proteins impart smooth muscles a contractile phenotype. The mechanism of ASMCs contraction includes the interactions of actin and myosin light chain proteins [59] and requires an increase in intracellular Ca^++^. The main source of this calcium is intracellular sarcoplasmic reticulum (SR) stores. The release of Ca^++^ from the SR involves the entry of inositol 1,4,5-trisphosphate (IP3) into the SR. The formation of IP3 starts from membrane-bound phosphatidylinositol 4-phosphate (PI4P), which after phosphorylation by PIP5K1γ gets converted into phosphatidylinositol 4,5-bisphosphate (PIP2). PIP2 is the precursor of soluble IP3 and this reaction is catalyzed by the enzyme phospholipase C (PLC), which generates membrane-bound residual di-acyl glycerol (DAG) and soluble IP3 from PIP2 [60].

Polyamines are well known for their effects on cell proliferation and viability. Airway remodeling in asthma requires ASMCs proliferation [57]. During proliferation, ASMCs change from the contractile to the synthetic phenotype. The role of polyamines in this context was speculated and the expression of ODC was found to be responsible for this transition in arterial smooth muscle cells, since inhibiting ODC using DFMO prevented this transition. In murine ASMCs, polyamines, specifically spermine, were shown to induce acetylcholine-mediated contraction in ex vivo cultures of ASMCs [61] (Figure 2). A possible effect of spermine in this contraction could be due to the reduction in the availability of nitric oxide (NO) for the relaxation of smooth muscles, since L-arginine is a common precursor for the synthesis of both polyamines as well as NO. Additionally, polyamines, mainly spermine, along with Mg^2+^ ions are potent inducers of PIP5K1γ in ASMCs, which is a determinant enzyme for the formation of the IP3 molecule, an inducer of the release of sarcoplasmic Ca^2+^ [62]. An interesting role of the polyamine catabolic enzyme SAT1 in smooth muscle contraction was published by Chen et al., who showed the intracellular interaction of SAT1 with a transmembrane protein α9β1 integrin is important for the smooth muscle relaxation [63]. This interaction was important for the degradation of larger polyamines, spermine and spermidine, to the smallest polyamine putrescine, which does not induce PIP5K1γ and thereby leads to the relaxation of smooth muscle contraction. In this study, treatment of isolated murine tracheal rings with BENSPM induced polyamine catabolism and led to the relaxation of methacholine (a bronchoconstrictor)-induced ASMCs; however, induction of SAT1 was not effective when α9β1 integrin was knocked out. In addition to in vitro findings in tracheal ASMCs, spermine nebulization to naïve mice was shown to induce airway hyperresponsiveness [14].

## 7. Discussion

Though polyamines are needed for cell survival and proliferation, if not maintained optimally, their altered levels may dispose a cell to various kinds of stress. Asthma is a complex disease which can be defined by chronic airway inflammation, remodeling and hyperresponsiveness; it is interesting to note that polyamines have been linked with all of these features. Polyamines have been found to be high in the circulation of asthmatics with active symptoms and in the lungs of murine model of experimental asthma [14,44]. Since polyamines have been found to be high in the circulation as well as in the lungs, it is pertinent that they may be involved in various molecular or cellular events, locally as well as systemically. Different well-designed studies have reported their roles in these scenarios.

In immune cells such as mast cells and neutrophils, polyamines increase the pathogenic potential by inducing an active state; in the case of mast cells, by imparting structural support to secretory granules and also by inducing the release of preformed inflammatory mediators; whereas in neutrophils, by enhancing the generation of ROS. While one study on mast cells, extracellular addition of polyamines enhanced histamine release [44], another report demonstrated that polyamines themselves were present in the secretory granules of mast cells [46]. Interestingly, polyamine-containing granules were lacking histamines, but depletion of polyamines after using DFMO resulted in an increased secretion of histamine, indicating a definite but incompletely explored role of polyamines in histamine storage and secretion from mast cells. The discovery of a mast cell polyamine transporter, VPAT [47], could be tested in an in vivo model of asthma or patient samples. Its potential to play an important role in asthma pathology could implicate it as an important therapeutic target.

With another granulocyte, the neutrophil, polyamines were found to potentiate the ROS generation mediated by FMLP [52]. However, this observation was not further explored in full detail and it was speculated that polyamines either increase the interaction of the bacterial peptide with neutrophils or enhance the entry of exogenous Ca^2+^. Moreover, this finding could be tested in severe asthmatic conditions, where a neutrophilic response is dominant and patients are resistant to existing steroid therapy.

Eosinophils from normal and asthmatic patients, when exposed to spermine-rich conditions, survive longer than their natural life span and increase the expression of the activation marker CD11b on their surface. Spermine was demonstrated to inhibit mPT-induced apoptosis of eosinophils along with caspase cascade activation [48]. This interesting observation could be tested further in the context of other granulocytes in asthma where polyamines may also show survival-promoting effects. Hence, a polyamine-rich environment may induce the active repertoire of inflammatory immune cells in asthma, which may further amplify this response with their active interactions with other immune cells.

In addition to enhancing the inflammatory potential of granulocytes, polyamines play a crucial role in the differentiation of monocytes towards the M2 phenotype in the presence of a Th2 cytokine of asthma [55]. This study highlighted the induction of several polyamine-dependent genes important for the M2 phenotype, whereas genes important for the M1 phenotype were repressed in the presence of polyamines. Earlier reports had shown the importance of polyamines in the modulation of gene expression by affecting the acetylation of histones, ultimately affecting the access of transcription factors to their binding sites on DNA [64,65]—a mechanism speculated for the polyamine-mediated alternative activation of macrophages. Interestingly, during *Helicobacter pylori* and *Citrobacter rodentium* infections, ODC was found to inhibit M1, while ODC inhibition promoted the M1 phenotype. Further, myeloid-specific deletion of ODC promoted the M1 phenotype, with the secretion of M1-specific cytokines and chemokines that resulted in the resolution of bacterial infection in mice [66]. However, this deletion did not promote the M2 phenotype in contrast to the previous reports, demonstrating that the presence or absence of polyamines can switch the macrophage phenotype to M1 or M2, respectively. Moreover, during *H. pylori* and *C*. *rodentium* infection, spermine and spermidine were not very effective in suppressing the M1 phenotype, however, putrescine played a decisive role in chromatin remodeling (for euchromatin transformation) and in inhibition of M1 differentiation.

These findings together suggest that the presence of polyamines is a determinant for differentiation towards the M2 phenotype in an environment-dependent manner. In the presence of M2-promoting factors like IL-4, polyamines act as essential cofactors for M2 polarization and simultaneously inhibit the gene expression of the M1 phenotype. However, another study demonstrated that during bacterial infection, depletion of polyamines is required for M1 polarization, but it does not promote the M2 phenotype in the absence of other required factors for M2 polarization. In conclusion, polyamines are cofactors for the differentiation towards M2 phenotype in the presence of a Th2-dominant environment, and the loss of either the polyamine anabolic enzyme ODC or polyamines, specifically putrescine, orchestrates the M1 response.

ASMCs are the crucial component of asthma-associated airway hyper-responsiveness and airway remodeling. In asthma, spermine could play an important role in the induction of ASMCs contraction and thus airway hyper-responsiveness, by either reducing the availability of NO for ASMCs relaxation [14] or by activating the PIP5K1γ enzyme in ASMCs [63]. While the interaction between the polyamine catabolic enzyme SAT1 and intracellular domain of α9β1 integrin was found to be important for the relaxation of ASMCs, SAT1 alone was not effective in reducing the local concentration of polyamines in the absence of integrin [63]. Hence, induction of SAT1 and restoration of reduced levels of α9β1 integrin, both could be effective in relaxation of ASMCs contractions in asthma. However, the role of SAT1 in asthma needs to be explored further.

The dysregulation of polyamine anabolism in asthma was thought to be an important driver for the high levels of polyamine found, and an attempt was made to bring down these high levels by inhibiting ODC using DFMO in an allergic acute model of asthma. Though DFMO could not bring down the high levels of spermine and the histological features of asthma, it could successfully reduce airway hyper-responsiveness in a murine asthma model [14]. In addition to their effects on airway mechanics, polyamines induce the expression of transforming growth factor beta (TGF-β) in intestinal epithelial cells [67]. TGF-β is an important cytokine for airway remodeling during asthma. It has multiple effects on different immune and structural cells of the lung and has an active role in pulmonary fibrosis [68]. Notably, spermine levels were reported high in the sputum of cystic fibrosis (CF) patients and were associated with pulmonary exacerbations. Moreover, treating CF patients with antibiotics improved bronchoconstriction with a reduction in the levels of spermine [61]. Additionally, spermine treatment to mouse bronchial rings enhanced acetylcholine-induced constriction. These findings in structural cells indicate a pathophysiological role of polyamines in asthma. In addition to polyamine metabolism, polyamine transport may also play a crucial role in asthma pathogenesis, but due to a lack of in-depth knowledge of polyamine transport in mammalian systems, this area requires research to reach the level of therapeutics.

The polyamine pathway seems to be a very promising target in asthma due to its involvement in the important processes which play crucial roles in multiple pathways of asthma pathogenesis. Since asthma is a complex disease, finding a common node involving polyamines which regulates multiple pathways of asthma could be beneficial.

## 8. Conclusions and Future Perspective

To the best of my knowledge, this review summarizes the key research reports to date, relevant to asthma and polyamines. The polyamine pathway seems a central node, which can regulate multiple arms of asthma pathogenesis. Though the effects of polyamines on immune and structural cells have been reported, in depth knowledge of polyamine involvement in asthma pathogenesis still requires further investigation. Different pharmacological modulators of the polyamine pathway could be tested in asthmatic conditions. Hence, positive outcomes from findings of the polyamine pathway in asthma provide an impetus to investigate the polyamine pathway in more detail in asthma.
